# Parvin Overexpression Uncovers Tissue-Specific Genetic Pathways and Disrupts F-Actin to Induce Apoptosis in the Developing Epithelia in *Drosophila*

**DOI:** 10.1371/journal.pone.0047355

**Published:** 2012-10-15

**Authors:** Maria Chountala, Katerina M. Vakaloglou, Christos G. Zervas

**Affiliations:** Division of Genetics, Biomedical Research Foundation of the Academy of Athens (BRFAA), Athens, Greece; Dresden University of Technology, Germany

## Abstract

Parvin is a putative F-actin binding protein important for integrin-mediated cell adhesion. Here we used overexpression of *Drosophila* Parvin to uncover its functions in different tissues *in vivo*. Parvin overexpression caused major defects reminiscent of metastatic cancer cells in developing epithelia, including apoptosis, alterations in cell shape, basal extrusion and invasion. These defects were closely correlated with abnormalities in the organization of F-actin at the basal epithelial surface and of integrin-matrix adhesion sites. In wing epithelium, overexpressed Parvin triggered increased Rho1 protein levels, predominantly at the basal side, whereas in the developing eye it caused a rough eye phenotype and severely disrupted F-actin filaments at the retina floor of pigment cells. We identified genes that suppressed these Parvin-induced dominant effects, depending on the cell type. Co-expression of both ILK and the apoptosis inhibitor DIAP1 blocked Parvin-induced lethality and apoptosis and partially ameliorated cell delamination in epithelia, but did not rescue the elevated Rho1 levels, the abnormal organization of F-actin in the wing and the assembly of integrin-matrix adhesion sites. The rough eye phenotype was suppressed by coexpression of either PTEN or Wech, or by knock-down of *Xrp1*. Two main conclusions can be drawn from our studies: (1), high levels of cytoplasmic Parvin are toxic in epithelial cells; (2) Parvin in a dose dependent manner affects the organization of actin cytoskeleton in both wing and eye epithelia, independently of its role as a structural component of the ILK-PINCH-Parvin complex that mediates the integrin-actin link. Thus, distinct genetic interactions of Parvin occur in different cell types and second site modifier screens are required to uncover such genetic circuits.

## Introduction

Epithelial tissue morphogenesis involves cell shape changes that are induced by tightly regulated interactions between adhesion proteins and the associated actin cytoskeleton. Thus, proteins that modify either the adhesive properties of cells or the dynamics of actin organization have profound effects on epithelial patterning. In pathological situations including cancer, abnormal protein expression drives cells to acquire metastatic properties and break the epithelial integrity.

Integrins comprise a major cell surface protein family that mediate cell adhesion with the extracellular microenvironment and their function is essential for several tissue morphogenetic events during development [1]. Inside the cell, integrins organize the assembly of a large protein network, the adhesome, which mediates linkage with the actin cytoskeleton [2]. Parvin is a core component of the integrin adhesome and binds directly to integrin-linked kinase (ILK). Members of the highly conserved Parvin protein family contain two tandem unconventional Calponin-Homology (CH)-domains [3]. In contrast to mammalian α, β and γ-*parvin*, invertebrates have a single *parvin* homolog [4]. Genetic data in mice have demonstrated the important role of Parvin in integrin-mediated adhesion and our previous genetic analysis in *Drosophila* revealed that Parvin is also essential for adhesion in muscle and wing epithelia [5], [6]. In addition to these developmental functions, recent studies have linked β-Parvin expression to tumor suppressor effects during breast cancer formation in mice [7]. Misexpression studies and modifier screens aimed at identifying genetic circuits regulated by Parvin are of great importance to elucidate the tissue-specific molecular functions of Parvin in the context of a whole organism.

Here we took advantage of the *Drosophila* system to determine the effects of high levels of Parvin at the cellular level in several tissues and to investigate the tissue-specific suppression or enhancement of these defects by specific genes.

**Figure 1:**
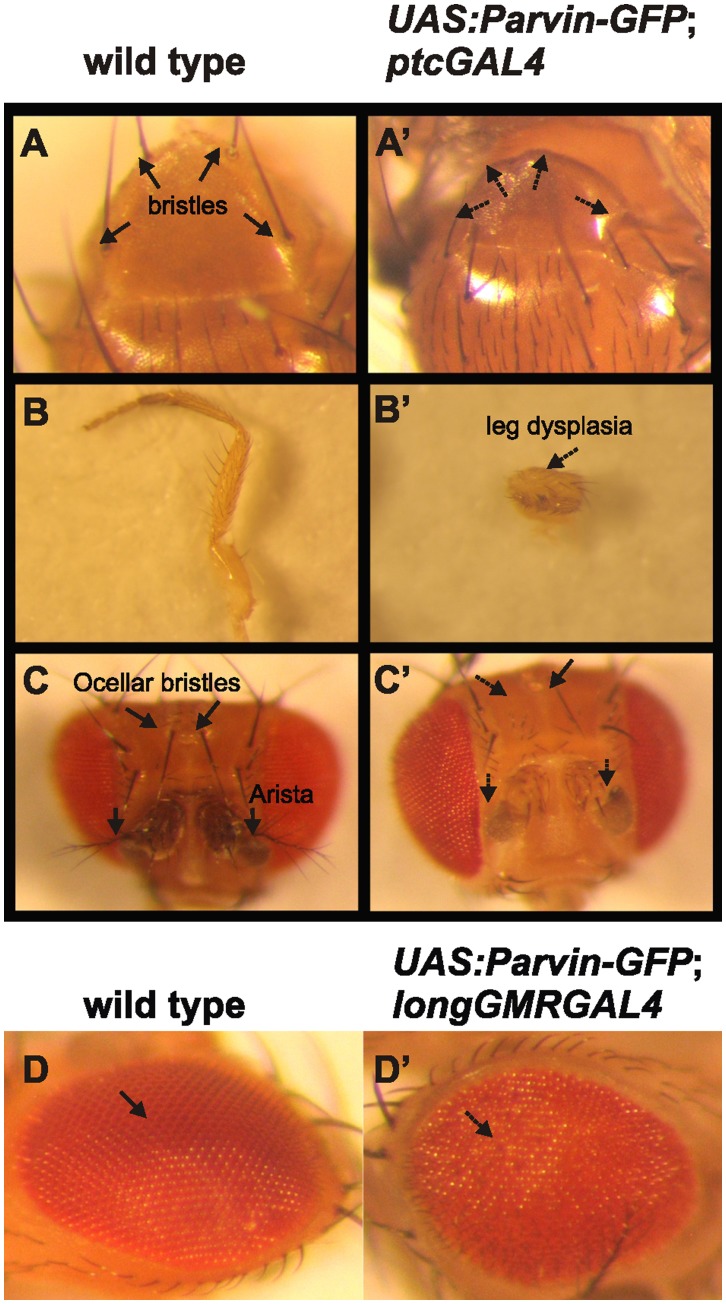
Overexpression of Parvin results in morphogenetic defects at various tissues in the adult fly. Images were collected with a cooled CCD camera for various adult structures. Thoracic bristles in wild type (A) were missing upon expression of *UAS::Parvin-GFP* under *ptcGal4* (A′). Leg from a wild type adult fly (B) was malformed when *UAS::Parvin-GFP* was expressed under *ptcGal4* (B′). Ocellar bristles and arista from the head of a wild type adult fly (C) were missing upon *UAS::Parvin-GFP* expression under *ptcGal4* (C′). A compound eye from a wild type adult fly (D) took on a rough appearance when *UAS::Parvin-GFP* was expressed under *longGMRGal4* (D′). Arrows depict a wild type tissue structure, whereas dashed arrows indicate defects.

**Table 1:** Gal4 drivers used to direct expression of *UAS::Parvin-GFP* during *Drosophila* development.

Gal4 driver	Tissue of expression	UAS::Parvin-GFP expression effect
*mef2*	mesoderm	larval lethality
*24B*	mesoderm and tendon cells	larval lethality
*drm*	gut and malpighian tubules	larval lethality
*48Y*	endoderm	loss of maxillary palpus
		segregation of cuticular vesicles
*elav*	nervous system	no phenotype
*sim*	ventral nerve cord	no phenotype
*69B*	epidermis and imaginal discs	mild rough eye
		missing posterior cross vein
*sev*	eye	no phenotype
*en*	epidermis and imaginal discs	pupae lethality
		tissue loss in the wing discs
*ptc*	epidermis and imaginal discs	loss of arista
		loss of ocellar bristles
		loss of thoracic bristles
		leg dysplasia
		mild rough eye
*longGMR*	eye	mild rough eye

## Materials and Methods

### Genetics and *Drosophila* Stocks

All transgenic strains encoding *UAS::Parvin* and its mutated forms were previously described [6]. Recombinant lines of *UAS::Parvin-GFP* with *longGMRGal4* were generated by standard meiotic recombination. In the eye modifier screen, virgin females of *w;;longGMRGal4, UAS::Parvin-GFP* were crossed with males of the tested strain from three different categories: (1) UAS lines expressing specific genes; (2) UAS::IR (RNAi-lines) derived either from the VDRC or the NIG collection; and (3) deficiencies included in the deficiency kit for the third chromosome derived from Bloomington. The following stocks were used: *UAS::Wech-GFP* (M. Hoch); *UAS::PTEN* (A. Manoukian and T. Xu); *puc-laZ* (S. Noselli); *UAS::ABDMoesinRFP* (T. Millard) and *UAS::DIAP1* (Bloomington); *UAS::ILK* [8]; *UAS::αPS1*; *UAS::αPS2*; and *UAS::βPS* (N. Brown). *Gal4* drivers were obtained from Bloomington. All crosses were performed at 25°C.

**Table 2:** Truncated forms of *UAS::Parvin-GFP* expressed with specific *Gal4* drivers did not affect tissue morphogenesis.

UAS Transgene	Gal4 drivers tested	Phenotypeinduced
UAS::Parvin^ΔCH2^-GFP	*mef2/24B/en/ptc/longGMR*	no
UAS::Parvin^ΔCH1^-GFP	*mef2/24B/en/ptc/longGMR*	no
UAS::Parvin^CH1LCH2^-GFP	*mef2/24B/en/ptc/longGMR*	no

**Figure 2:**
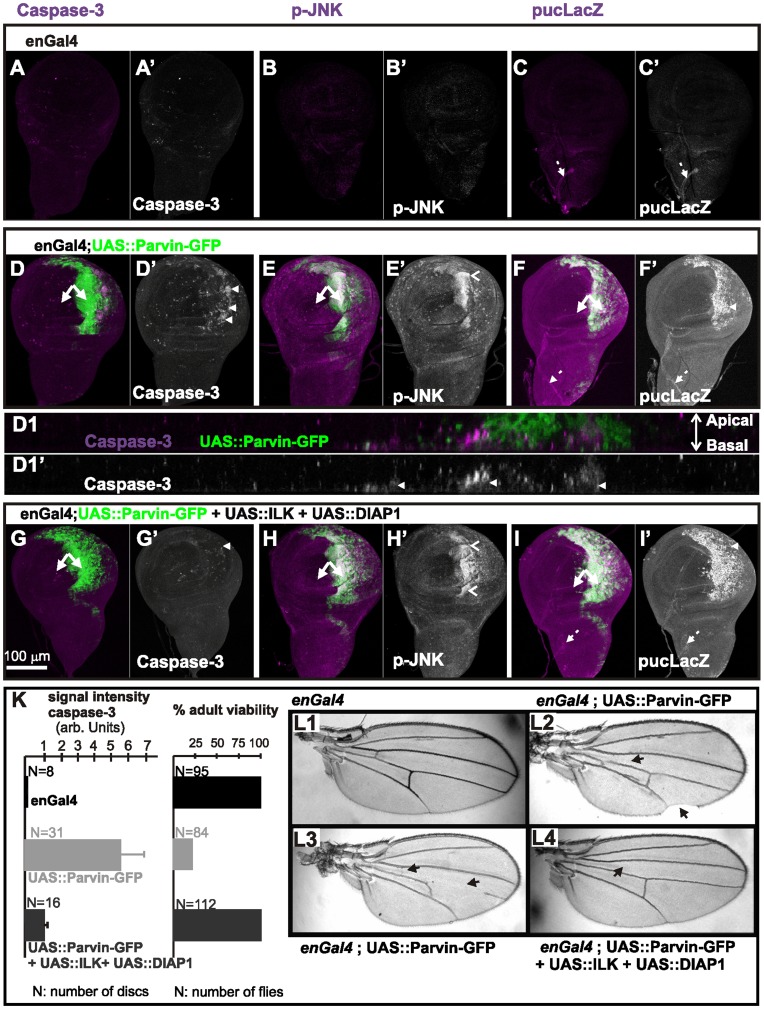
Parvin overexpression induces apoptosis and activation of JNK signaling. (A–I) Wing imaginal discs of late third instar larvae from control *enGal4* (A–C), *enGal4/+;UAS::Parvin-GFP/+* (D–F), *enGal4/UAS:ILK;UAS::Parvin-GFP/UAS:DIAP1* (G–I). Wing discs expressed either only Gal4 (A–C), or Parvin-GFP (green, D–F), or ILK, DIAP1 and Parvin-GFP together (green, G–I), and were probed for activated caspase-3 (magenta, A, D, G; white A′, D′, G′), active p-JNK (magenta, B, E, H; white B′, E′, H′), or lacZ expressed from the *puc* locus (magenta, C, F, I; white C′, F′, I′). (D1–D1′) A cross optical section taken in the middle of the wing poutch from the imaginal disc appearing in image D. Arrowheads indicate apoptotic cells; open arrowheads indicate areas of active JNK; arrows, closed areas in the posterior and anterior compartment of the wing pouch expressing (right) or not expressing (left) *UAS::Parvin-GFP*; dashed arrows indicate stalk cells in the wing notum area. The anterior part of the wing disc (where Parvin-GFP expression is not induced) serves as an internal control. (K) Quantification of caspase-3 signal intensity in wing imaginal discs and the percentage of adult viability. (L1–L4) Adult wings derived from flies expressing only *enGal4* (L1), *UAS::Parvin-GFP* under *enGal4* (L2, L3) and *UAS:ILK* together with *UAS:DIAP1* and *UAS::Parvin-GFP* under *enGal4* (L4). Arrows indicate vein defects and notched areas in the wing.

**Figure 3:**
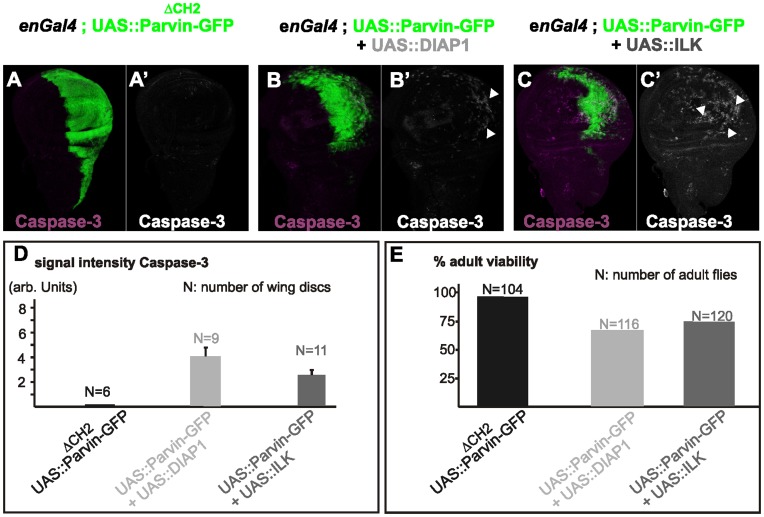
Expression of Parvin lacking the CH2-domain and Parvin coexpression with ILK or DIAP1. Confocal optical sections acquired from wing imaginal discs of late third instar larvae expressing *UAS::Parvin^ΔCH2^-GFP* (green, A), or coexpressing either *UAS:DIAP1* and *UAS::Parvin-GFP* (green, B) or *UAS:ILK* and *UAS::Parvin-GFP* (green, C), under *enGal4* in the posterior compartment of the disc, probed for activated caspase-3 (magenta, A–C; white A′–C′). Arrowheads indicate apoptotic cells. The anterior part of the wing disc (where Parvin-GFP expression is not induced) serves as an internal control. (D–E) Quantification of the caspase-3 signal intensity in wing imaginal discs (D) and the percentage of adult viability (E).

### Immunohistochemistry and Confocal Microscopy

Eye and wing discs were dissected from third-instar larvae or 75% pupae and fixed according to standard protocols [6], [8]. Primary antibodies were against: active caspase-3 (1∶250, Cell Signaling); active JNK (1∶500, Cell Signaling); MMP1 (1∶50, mix in 1∶1∶1 of 5H7B11/3A6B4/3B8D12, DSHB); βPS-integrin (1∶10, CF.6G11, DSHB); Ena (1∶50, 5G2, DSHB); Cadherin (1∶50, DCAD2, DSHB); Rho1 (1∶50, p1D9, DSHB); LamininA (1∶500, [9]) and Dia (1∶250, provided by S. Wasserman, UCSD, USA). F-actin was labelled using either rhodamine or Alexa-Fluor-633 phalloidin (Molecular Probes). Secondary antibodies were used at a dilution of 1∶500 and were conjugated to Alexa-Fluor-488, -568, or -633 (Molecular Probes). Nuclei were labelled with DAPI. Images were obtained with a Leica SP5 confocal microscope, using the 20X/0.7NA objective or an oil 63X/1.4 NA objective. Leica SP5 software was used for quantitative analysis of the immunolabelled tissues. The compared images were acquired with identical settings of laser power, gain and iris while avoiding saturation of pixel intensity. Selected areas were outlined and the total intensity was measured and plotted using Excel. Images from adult eyes were obtained using either a Leica DFC500 cooled CCD camera or a Leica TCS LSI system. All images were assembled in Photoshop 7 and labelled in Corel Draw 12.

**Figure 4:**
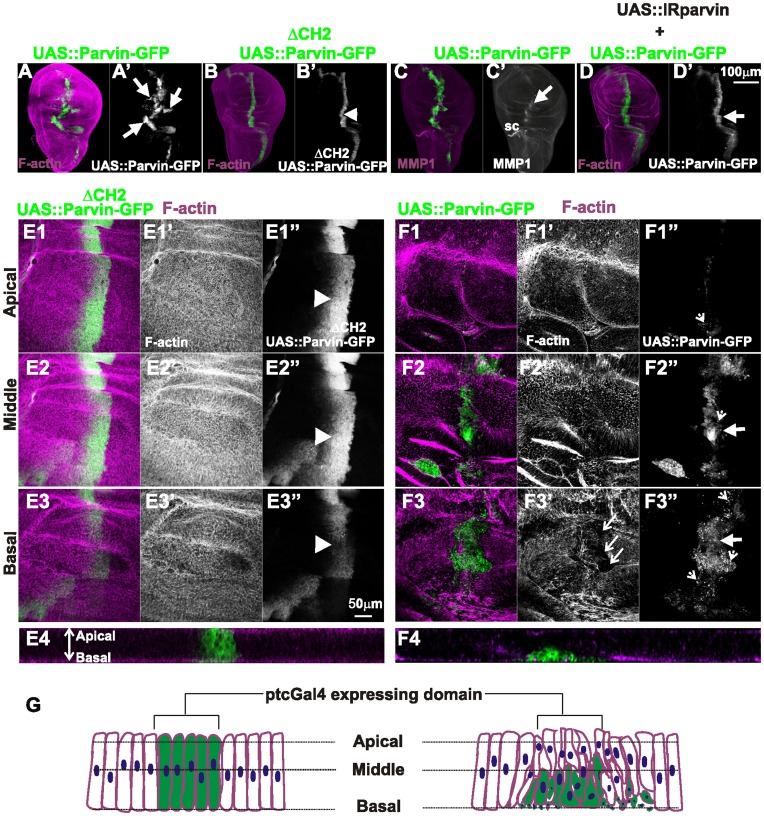
Parvin overexpression in the wing epithelium leads to cell extrusion, MMP1 secretion and invasion. Confocal projections of wing imaginal discs of late third instar larvae (A–D), and optical sections acquired at different focal planes as indicated (E1, E2, E3, E4–F1, F2, F3, F4), with *ptcGal4* driving expression of *UAS::Parvin-GFP* (A, C, F1–F4), or *UAS::Parvin^ΔCH2^-GFP* (B, E1–E4), or *UAS::Parvin-GFP* together with *UAS:IRParvin* (D). Imaginal wing discs expressing Parvin-GFP (green, A–F3; white, A′–D′, E1′′–F3′′) were probed for rhodamine-phalloidin to visualize F-actin (magenta, A–B, D, E1–E4, F1–F4; white, E1′–E3′, F1′–F3′) or MMP1 (magenta, C; white C′). (E4–F4) Cross optical sections of the imaginal discs appearing in images E1–F1 taken in the middle of the wing poutch.Graphic cartoon based on the optical sections shown in images E4 and F4 (G). Arrowheads indicate the expression domain of *ptcGal4* that drives expression of the transgene *UAS::Parvin^ΔCH2^-GFP*; large arrows point to migrating cells that have invaded the proximal areas of *UAS::Parvin-GFP* expression; dashed arrowheads denote small cellular debris containing pyknotic nuclei; and open arrows show areas lacking F-actin staining in the basal side of discs expressing *UAS::Parvin-GFP.* sc; stalk cells.

## Results

### Parvin Overexpression during Development Causes Morphogenetic Defects

In mammalian cells, α-Parvin has an anti-apoptotic function whereas β-Parvin promotes apoptosis [10], [11]. We followed a gain-of function approach utilizing the *UAS/Gal4* system [12] to overexpress Parvin in several tissues during development (Table 1). We focused mainly on the wing epithelium and the eye, using *ptcGal4, enGal4* and *longGMRGal4* drivers. Overexpression of Parvin by *ptcGal4* resulted in several abnormal developmental defects including loss of thoracic bristles, dysplasia in legs, loss of arista and ocellar bristles in the head, whereas a fraction of flies died during pupae development (Figure 1A′–C′). Parvin overexpression driven by *longGMRGal4* caused a rough eye phenotype (Figure 1D′). Finally, induction of Parvin expression with *enGal4* mostly caused lethality, while the surviving flies had wing defects (Figure 2L2, L3). Fly morphogenesis was not interrupted by similar levels of overexpression of several domain deletion UAS::Parvin-GFP constructs (Table 2), suggesting that combinatorial interactions of Parvin domains are required to elicit a lethal effect and that only high levels of full-length Parvin are detrimental for the whole organism.

**Figure 5:**
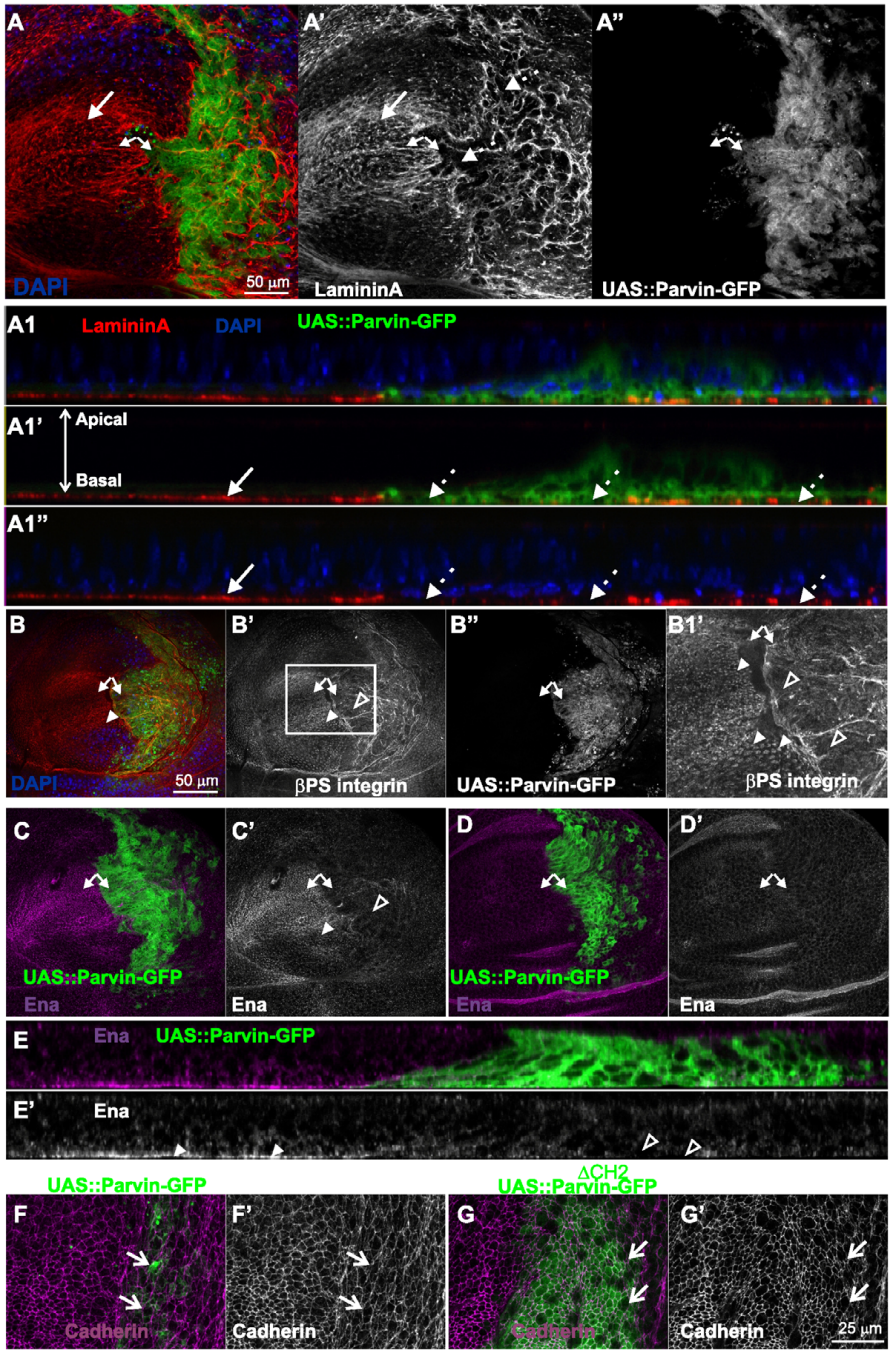
Parvin overexpression in the wing epithelium disrupts basal adhesion without affecting apicobasal polarity. Confocal optical sections acquired from wing imaginal discs of late third instar larvae expressing *UAS::Parvin-GFP* (green, A–F; white, A′′–B′′) or *UAS::Parvin^ΔCH2^-GFP* (green, G) under *enGal4* in the posterior compartment of the disc, probed for laminin (red, A–A1′′; white A′), βPS integrin (red, B; white B′, B1′), Ena (magenta, C–E; white C′–E′), and Cadherin (magenta, F–G; white F′–G′). Panels C–D show two different focal planes: basal (C), and intermediate (D) sides of the epithelium. (A1–A1′′, E–E′) cross optical sections of the imaginal discs appearing in images A and C respectively taken in the middle of the wing poutch. Small arrows, closed areas in the posterior and anterior compartment of the wing pouch expressing (right) or not expressing (left) *UAS::Parvin-GFP*; big arrows, indicate areas with LamininA deposition; big dashed arrows indicate areas without LamininA deposition; open arrows, apical boundaries of wing epithelial cells; arrowheads, focal contact-like structures in the basal side of the anterior compartment; open arrowheads, areas lacking integrin-matrix adhesion sites. The anterior part of the wing disc (where Parvin-GFP expression is not induced) serves as an internal control.

### Parvin Overexpression in the Wing Epithelium Leads to Apoptosis and Activation of the JNK Pathway

The morphogenetic defects caused by Parvin-GFP overexpression driven by *enGal4* suggested a pro-apoptotic function for Parvin in *Drosophila*, similar to β-Parvin in mammalian cells [11]. To further verify if Parvin-GFP overexpression caused apoptosis, we examined the levels of active Caspase-3. Active Caspase-3 was undetectable in control *enGal4* wing discs (Figure 2A, A′) or those expressing a CH2-domain deletion Parvin mutant fused to GFP (UAS::Parvin^ΔCH2^-GFP) (Figure 3A). In contrast, Parvin-GFP overexpression induced a large increase in active Caspase-3, specifically in the posterior compartment of the wing disc, compared to only a few apoptotic cells in the anterior compartment which serves as an internal control (Figure 2D, D1′, K). We used a commercially available antibody against active Caspase-3 that was recently reported to recognize not only Caspase-3 but also additional substrates cleaved in a *Drosophila* Nedd2-like caspase (DRONC)-dependent manner [13]. Thus, we concluded that Parvin-GFP overexpression induced elevation of the Caspase-9-like initiator DRONC that resulted in apoptosis.

**Figure 6:**
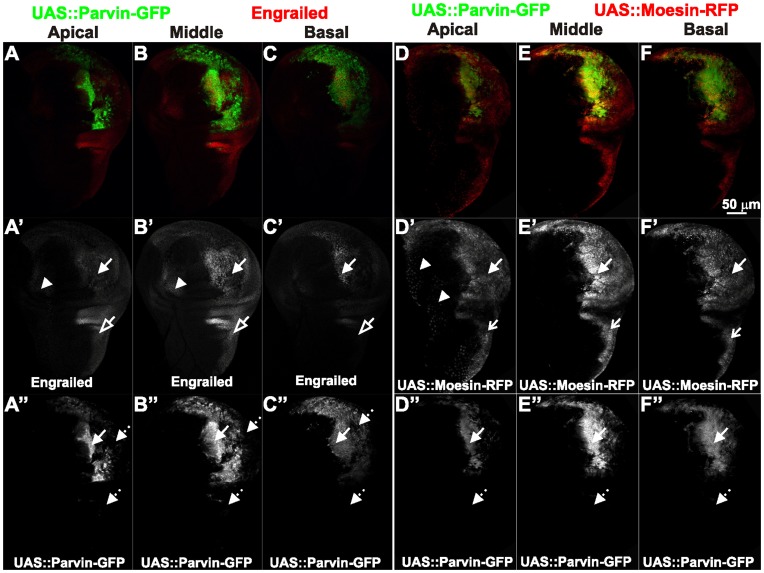
Posterior identity is retained in wing epithelial cells overexpressing Parvin. Confocal optical sections were acquired at different focal planes (A, D) apically, (B, E) in the middle, (C, F) at the basal side of wing imaginal discs of late third instar larvae expressing *UAS::Parvin-GFP* (green, A–C; white, A′′–C′′) and probed with antibodies against Engrailed (red, A–C; white A′–C′) or coexpressing *UAS:ABDmoesin-RFP* (red, D–F; white D′–F′) and *UAS::Parvin-GFP* (green, D–F; white D′′–F′′) under *enGal4* in the posterior compartment of the disc. Arrows: areas in the posterior compartment where expression of *UAS::Parvin-GFP* is retained; dashed arrows: areas in the posterior compartment that have lost expression of *UAS::Parvin-GFP*; arrowheads: cells in the anterior compartment expressing *enGal4*; open arrowheads: areas in the posterior compartment expressing Engrailed; open arrows: areas in the posterior compartment expressing *UAS:ABDmoesin-RFP*.

**Figure 7:**
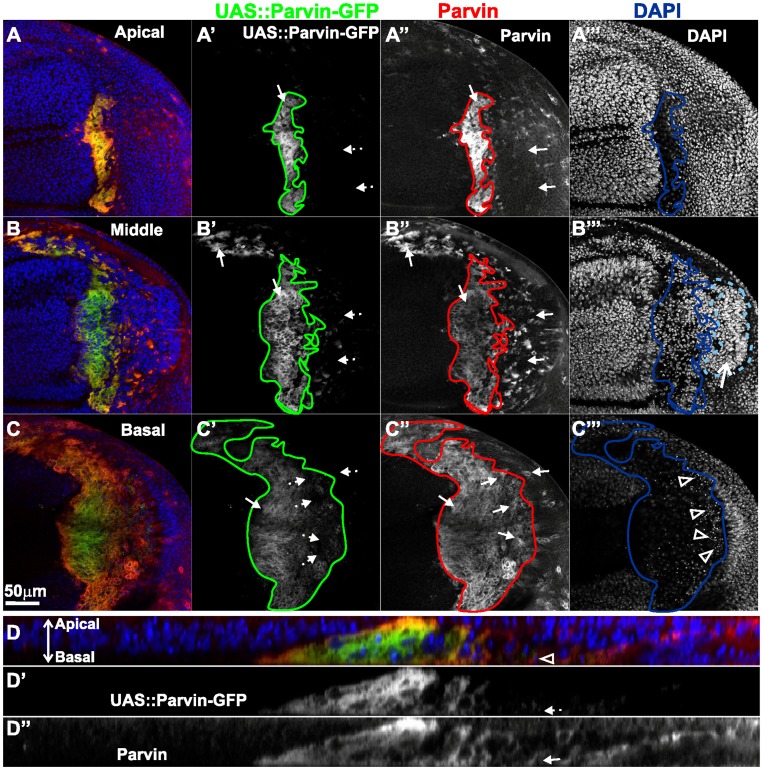
Comparison of anti-Parvin and Parvin-GFP detection in the wing imaginal discs. Confocal optical sections acquired from wing imaginal discs of late third instar larvae expressing *UAS::Parvin-GFP* (green, A–D; white A′–D′) under *enGal4* in the posterior compartment of the disc, probed with an antibody against Parvin (red, A–D; white A′′–D′′) and DAPI to visualize the nuclei (blue, A–D; white A′′′–C′′′). (D) cross optical section taken in the middle of the wing poutch from the imaginal disc appearing in images A–C. Arrows: cells expressing Parvin detected either by the antibody or by Parvin-GFP; dashed arrows: cells where Parvin-GFP is undetectable; open arrowheads:pyknotic nuclei; open arrow: an area in the posterior compartment with high density of nuclei outlined with a light blue dashed line.

Apoptotic stimuli are known to activate JNK signaling at the imaginal discs [14]. We examined whether Parvin–induced apoptosis is mediated by the JNK pathway, by immunostaining for the phosphorylated active form of JNK. The *Drosophila* homolog of JNK, *basket*, was highly phosphorylated specifically at the posterior compartment of the wing disc (Figure 2E–E′), compared to low levels of active JNK in control discs (Figure 2B–B′). We used the downstream target, *puckered*, as another marker for activation of the JNK pathway [15]. Cells ectopically expressing Parvin-GFP strongly upregulated the *puc-lacZ* reporter in the posterior compartment (Figure 2F–F′), whereas in control discs, *puc-lacZ* was detected only in the stalk cells (Figure 2C–C′). Thus, JNK signaling was activated by increased levels of Parvin-GFP within the wing imaginal disc.

**Figure 8:**
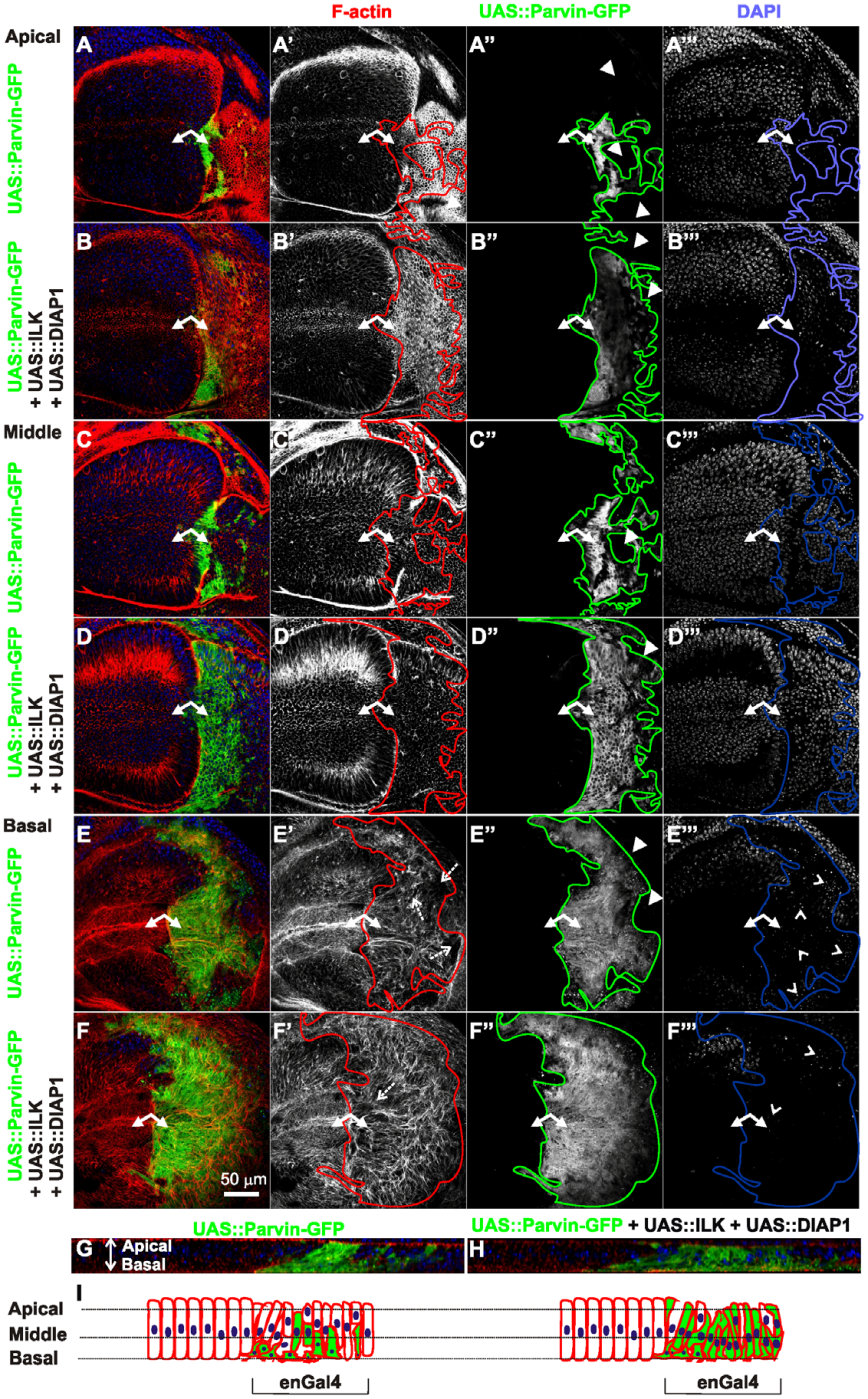
Parvin overexpression in the wing imaginal disc epithelium disrupts the basal F-actin cytoskeleton. Confocal optical sections acquired apically (A–B), in the middle (C–D) and at the basal side of the epithelium (E–F) from wing imaginal discs with *enGal4* driving expression in the posterior compartment of *UAS::Parvin-GFP* alone (green, A, C, E; white, A′′, C′′, E′′) or coexpression with *UAS:ILK* and *UAS:DIAP1* (green, B, D, F; white B′′, D′′, F′′). Imaginal discs were probed with rhodamine-labelled phalloidin to visualize F-actin (red, A–F; white A′–F′) and DAPI to visualize nuclei (blue, A–F; white A′′′–F′′′). (G) A cross optical section of the imaginal disc appearing in images A, C, and E. (H) a cross optical section of the imaginal disc appearing in images B, D, and F. (I) Graphic cartoon based on the cross optical sections G and H. Small arrows, closed areas in the posterior and anterior compartment of the wing pouch expressing (right) or not expressing (left) *UAS::Parvin-GFP*; Arrowheads, areas within the posterior compartment that do not express *UAS::Parvin-GFP*; dashed arrows, disrupted F-actin; open arrowheads, pyknotic nuclei. Lines encircle the region of the posterior compartment retained expression of *UAS::Parvin-GFP*. The anterior part of the wing disc (where Parvin-GFP expression is not induced) serves as an internal control.

**Figure 9:**
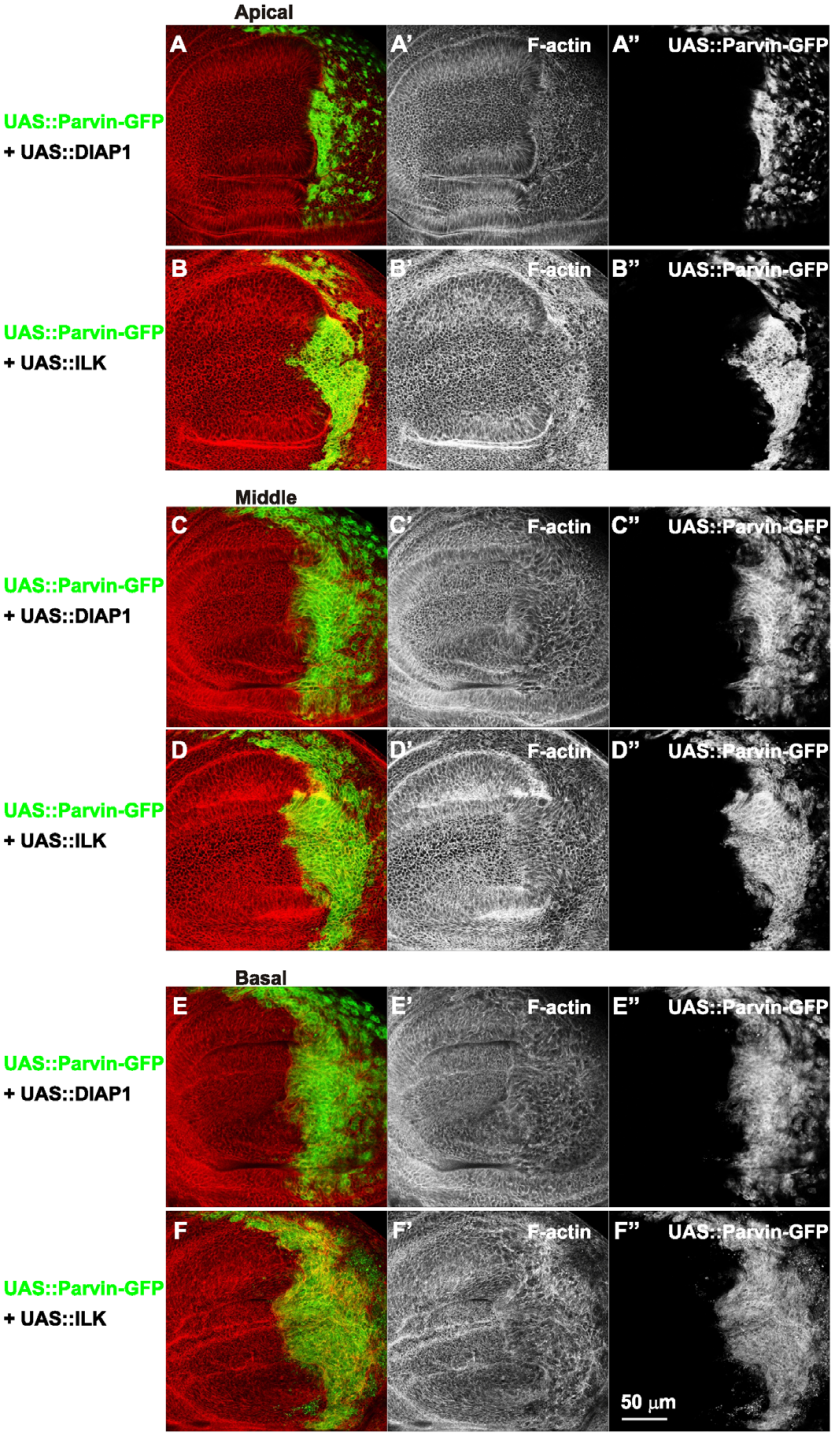
ILK and DIAP1 individually coexpressed with Parvin partially ameliorate F-actin misorganization in the wing epithelium. Confocal optical sections were acquired apically (A–B), in the middle (C–D) and on the basal side of the epithelium (E–F) from wing imaginal discs with coexpression driven by *enGal4* in the posterior compartment of *UAS::Parvin-GFP* (green, A–F; white A–F′′) and *UAS:DIAP1* (A, C, E) or *UAS::Parvin-GFP* and *UAS:ILK* (B, D, F). Imaginal discs were probed with rhodamine-labelled phalloidin to visualize F-actin (red, A–F; white A′–F′).

**Figure 10:**
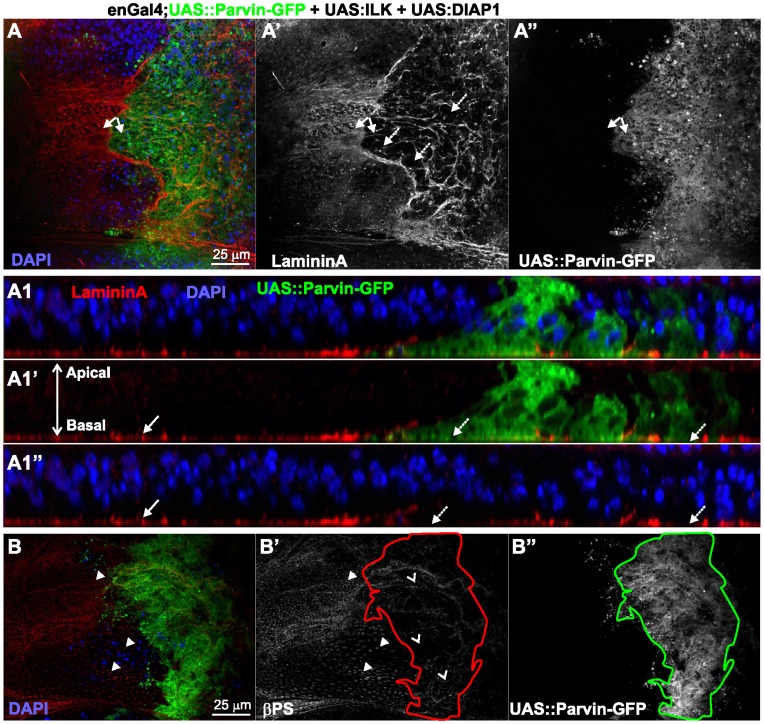
ILK and DIAP1 coexpressed with Parvin-GFP do not rescue the dissorganized integrin-matrix adhesion sites. Confocal optical sections acquired from wing imaginal discs of late third instar larvae expressing *UAS::Parvin-GFP* (green, A–B, A1–A1′; white, A′′–B′′) with both *UAS:ILK* and *UAS:DIAP1* driven by *enGal4* in the posterior compartment and probed for LamininA (red, A, A1–A1′; white, A′) or βPS integrin (red, B; white, B′) and DAPI to visualize nuclei (blue, A, A1, A1′′). (A1) cross optical section of the imaginal disc appearing in image A taken in the middle of the wing poutch. Small arrows, closed areas in the posterior and anterior compartment of the wing pouch expressing (right) or not expressing (left) *UAS::Parvin-GFP*; big arrows, indicate areas with LamininA deposition; big dashed arrows indicate areas without LamininA deposition; arrowheads, focal contact-like structures in the basal side of the anterior compartment and open arrowheads indicate areas in the posterior compartment without integrin accumulation. The anterior part of the wing disc (where Parvin-GFP expression is not induced) serves as an internal control.

### Increased Levels of Both ILK and DIAP1 Suppress Parvin-induced Apoptosis

Overexpression of Parvin-GFP driven by *enGal4* resulted in lethality mainly during pupae development. Only 20% of the late pupae developed into adult flies (Figure 2K) which exhibited various developmental defects in the wings, including tissue loss and vein defects (Figure 2L2, L3). To dissect the molecular mechanism of UAS::Parvin-GFP-induced apoptosis, we coexpressed Parvin-GFP with either ILK, a binding partner of Parvin, or *Drosophila* Inhibitor of Apoptosis Protein (DIAP1) [16]. Coexpression of DIAP1 alone largely suppressed the Parvin-GFP-induced dominant lethality and apoptosis (73% rescue of adult viability, n = 116) (Figure 3B, D, E). Coexpression of ILK [8] was less efficient at reducing the activation levels of DRONC, but significantly rescued lethality (75% rescue of adult viability, n = 120), similarly to DIAP1 expression alone (Figure 3C–E). However, coexpression of both ILK and DIAP1 completely blocked UAS::Parvin-GFP-induced apoptosis (Figure 2G–G′) and lethality (100% rescue of adult viability, n = 104) (Figure 2K), and the adult flies displayed only loss of posterior cross-veins (Figure 2L4). DIAP1 coexpression appeared to block Parvin-GFP-induced apoptosis at the final step of the process, because JNK signaling remained active in cells overexpressing both Parvin-GFP and DIAP1 (Figure 2H, I).

**Figure 11:**
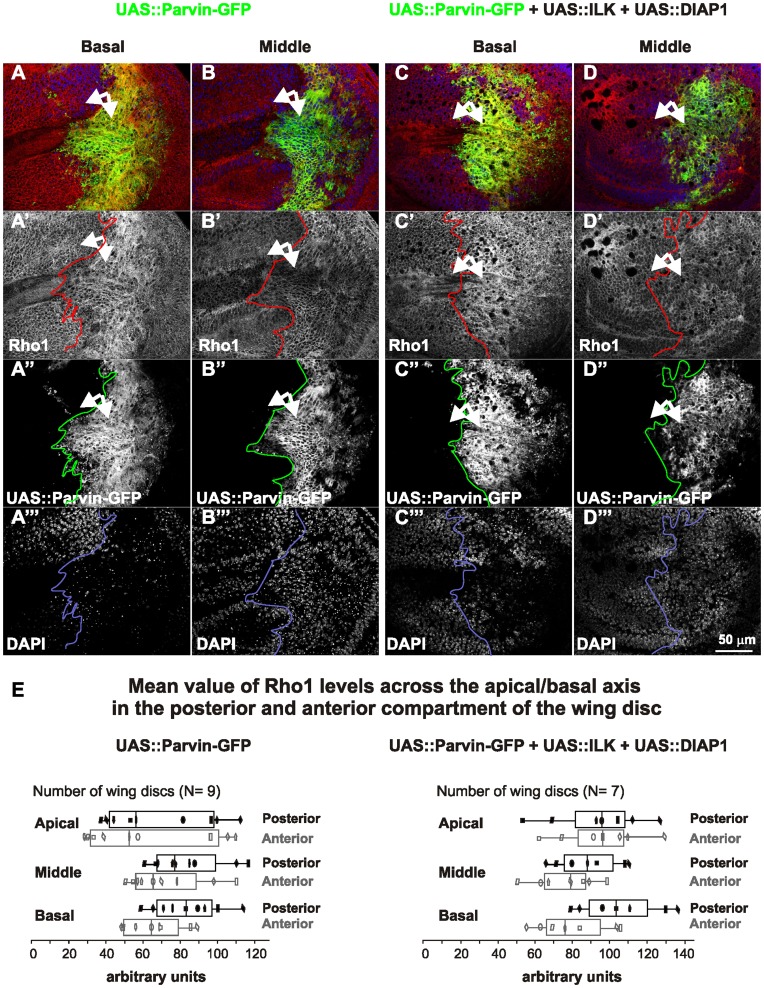
Parvin overexpression in the wing epithelium leads to Rho1 elevation, mainly basally. Confocal optical sections were acquired basally (A, C) or in the middle (B–D) from wing imaginal discs expressing *UAS::Parvin-GFP* alone (green, A, B; white, A′′, B′′) or with both *UAS:ILK* and *UAS:DIAP1* (green, C, D; white C′′, D′′), as driven by *enGal4* in the posterior compartment, probed for Rho1 (red, A–D; white A′–D′) and stained with DAPI to visualize nuclei (blue, A–D; white A′′′–D′′′). (E) Box-and-whisker plot of Rho1 levels indicating the means (vertical lines in the middle of the rectangular boxes) of measurements taken in apical, middle and basal focal planes. All individual measurements are superimposed on the box-and-whisker plots and are indicated by the same symbol in all focal planes to allow direct comparisons of the variation in pixel intensity. Arrows, closed areas in the posterior and anterior compartment of the wing pouch expressing (right) or not expressing (left) *UAS::Parvin-GFP*. The anterior part of the wing disc serves as an internal control.

**Figure 12:**
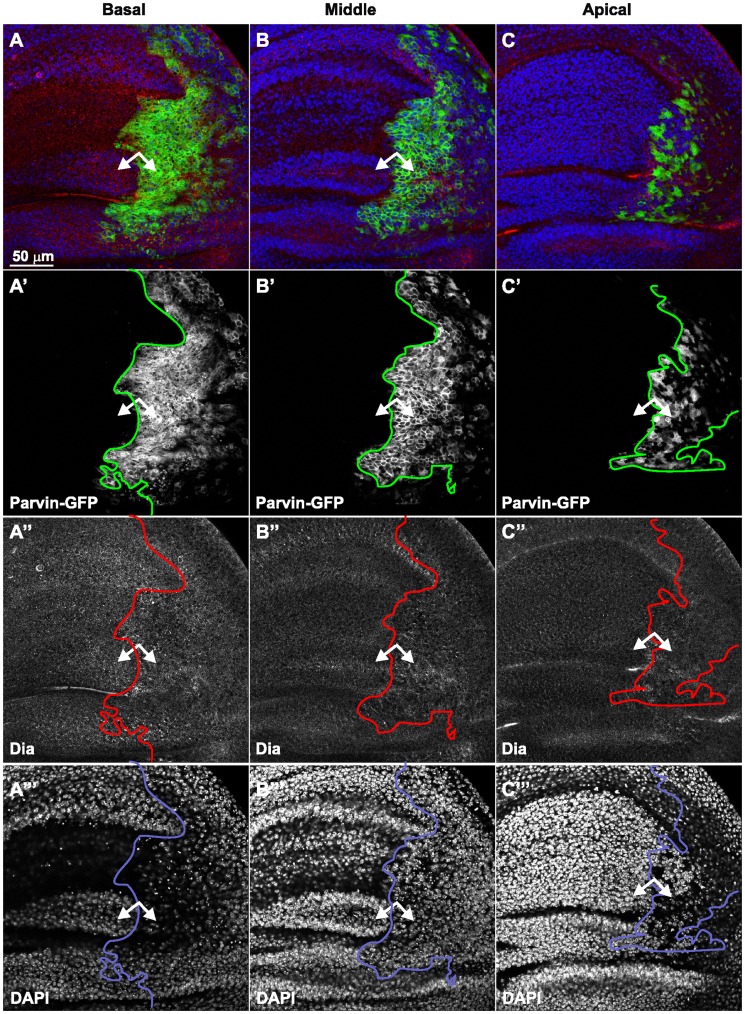
Parvin overexpression does not affect the endogenous levels of Dia. Confocal optical sections acquired basally (A), in the middle (B) or apically (C) of the wing imaginal discs with *enGal4* driving expression in the posterior compartment of *UAS::Parvin-GFP* (green, A–C; white, A′–C′) probed for the Rho1 effector Dia (red, A–C; white A′′–C′′) or DAPI to visualize the nuclei (blue, A–C; white A′′–C′′). Small arrows, closed areas in the posterior and anterior compartment of the wing pouch expressing (right) or not expressing (left) *UAS::Parvin-GFP*. The anterior part of the wing disc (where Parvin-GFP expression is not induced) serves as an internal control.

### Parvin Overexpression in the Wing Epithelium Leads to Cell Delamination, Cell Invasion and MMP1 Secretion

To further investigate the cellular consequences of Parvin-GFP overexpression, we used *ptcGal4* to drive expression in a thin stripe of cells anterior to the anteroposterior (A/P) boundary of the wing disc. Overexpression of UAS::Parvin-GFP triggered cell invasion in areas proximal to *ptcGal4* expression domain (Figure 4A). In contrast, overexpression of UAS::Parvin^ΔCH2^-GFP that was expressed even at higher levels than full-length Parvin-GFP [6] did not cause epithelial morphogenetic defects, indicating that the invasive phenotype was not a consequence of protein overexpression in general (Figure 4B). High magnification optical sections along the apical/basal side of the wing pouch revealed a large reduction in Parvin-GFP expressing cells, whereas cells expressing UAS::Parvin^ΔCH2^-GFP were maintained within the *ptcGal4* domain (Figure 4E1–F1). The UAS::Parvin-GFP expressing cells were extruded toward the basal side of the epithelium, where they acquired invasive properties that render them capable of migrating to the basal side of the epithelium and spread distant from the *ptcGal4* expression domain (Figure 4E2, E3, E4, F2, F3, F4, G). Several cells displayed small pyknotic nuclei indicative of apoptosis (Figure 4F3′′). Cell invasion was consistent with ectopic induction of matrix metalloproteinase-1 (MMP1) along the *ptcGal4* expressing region (Figure 4C). MMP1 is a well established effector of cell invasion that is upregulated upon JNK activation and is normally expressed only in the stalk cells of the wing disc (Figure 4C) [17].

**Figure 13:**
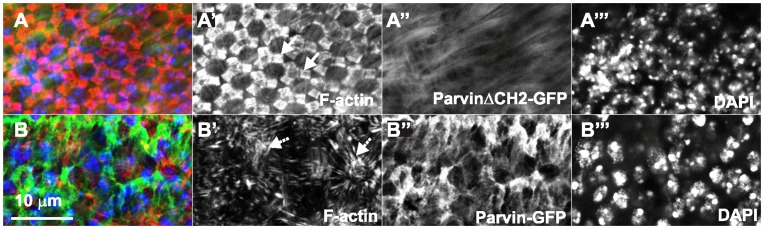
Parvin overexpression in the eye pigment cells disrupts F-actin cytoskeleton basally. Confocal optical sections acquired basally, in developing pupae eyes expressing control *UAS::Parvin^ΔCH2^-GFP* (A, green; A′′, white), or *UAS::Parvin-GFP* (B, green; B′′, white) with *longGMRGal4*, probed with rhodamine-phalloidin to visualize F-actin (red, A–B; white A′–B′) and stained with DAPI to visualize the nuclei (blue, A–B; white A′′′–B′′′). The pattern of F-actin in the retina floor is illustrated with the arrow.

To verify that a threshold level of UAS::Parvin-GFP is required to induce the invasive cell behavior, we coexpressed a UAS::RNAi construct known to knock down Parvin [6]. The moderate levels of Parvin-GFP expression along the *ptcGal4* domain did not cause migration of these cells distant from their original position (Figure 4D).

**Figure 14:**
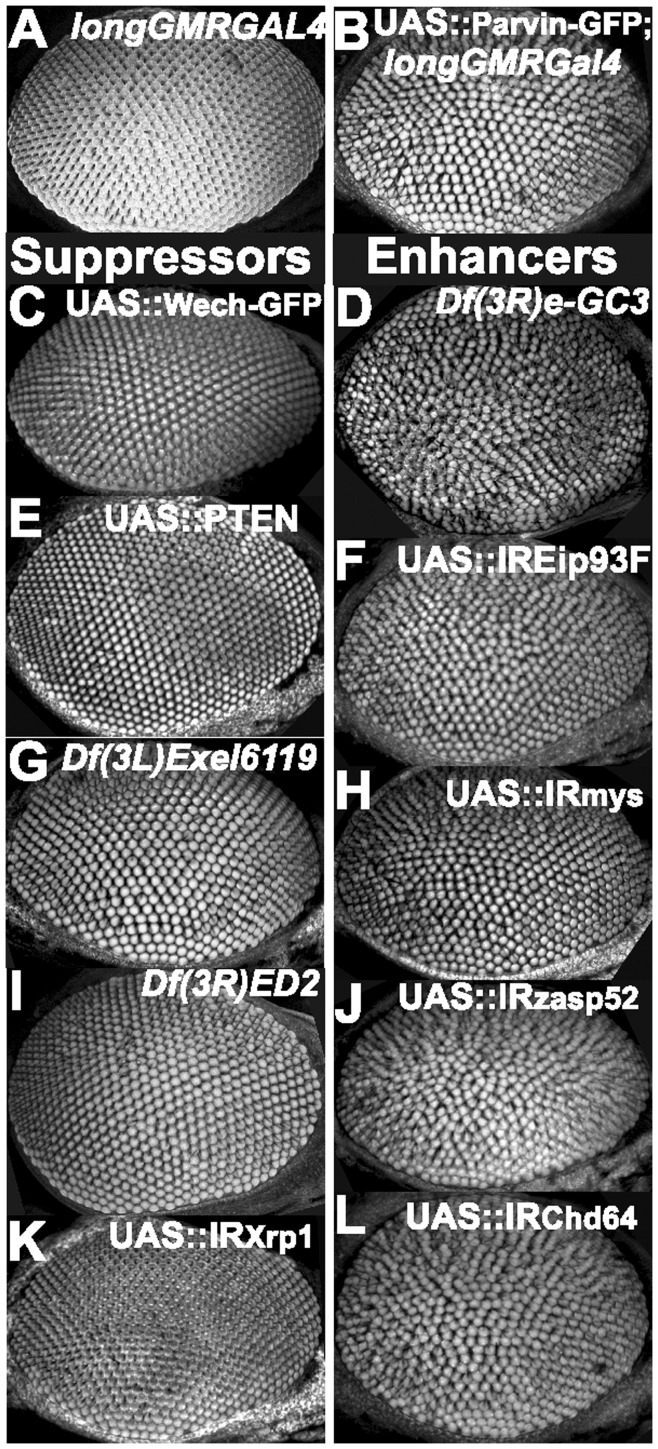
Rough eye phenotype and identification of enhancers and suppressors of *UAS::Parvin-GFP, longGMRGal4*. Adult eye confocal optical projections from the *longGMRGal4* stock served as control (A), the starting *UAS::Parvin-GFP, longGMRGal4* genotype (B) and the recovered modifiers (C–L). The main suppressors of the modifier screen show morphology of the ommatidia that is reminiscent of the control: *UAS:Wech* (C), *UAS:PTEN* (E), *Df(3L)Exel6119* (G), *Df(3L)ED2* (I), and *UAS:IRXrp1* (K). The main enhancers of the screen are *Df(3R)e-GC3* (D), *UAS:IREip93F* (F), *UAS:IRmys* (H), *UAS:IRzasp52* (J), and *UAS:IRChd64* (L).

### Parvin Overexpression in the Wing Epithelium Results in Loss of Cell-matrix Adhesion and Extracellular Matrix Disassembly without Affecting Cadherin Levels

In the wing imaginal discs integrin localizes largely in clusters containing adhesome proteins on the basal side of the epithelium, resembling the focal adhesions of mammalian cells [6], [18]. The ectopic elevated levels of MMP1 upon UAS::Parvin-GFP overexpression, prompted us to further investigate cell-matrix adhesion organization. LamininA, is a major component of the extracellular matrix (ECM) and it has been shown to localize basaly in the wing disc, where it displays a fibrillar distribution [19]. We found that overexpression of Parvin caused disorganization of LamininA in the posterior compartment of the wing epithelium. LamininA was reduced in certain areas and accumulated in others, displaying a non-ordered pattern of distribution (Figure 5A, A1–A1′′). Similarly the typical punctuate integrin localization at the focal contact-like structures at the basal side of the wing epithelium was severely affected, specifically in the posterior compartment, whereas large areas of the basal epithelium lacked integrin deposition (Figure 5B). Enabled (Ena) plays a role in the elongation of F-actin barbed end filaments and recently it was shown that is expressed in the wing disc [20], [21]. We found that within the anterior compartment, Ena accumulated basally at the focal-contact like structures, similarly to integrins and other integrin adhesome proteins [6], [18], whereas in the posterior compartment expressing UAS::Parvin-GFP, Ena was largely diminished (Figure 5C, E–E′). In contrast, in the middle and apical areas of the disc, Ena distribution was not affected, suggesting that its basal reduction was most likely a consequence of disorganized cell-matrix adhesion sites (Figure 5D–D′). Thus, we concluded that in the basal wing epithelium high levels of Parvin-GFP disrupt integrin-matrix adhesion sites.

**Table 3:** Summary of all identified modifiers of *UAS::Parvin-GFP, longGMRGal4* in the developing eye, including information on the stock used, the cytogenetic map for the deficiencies and the effect of each modifier.

Gene/Deficiency	Reference/stock	Cytogeneticmap	Effect
*UAS::β_PS_;UAS::α_PS1_*	[31]		**E**/mr
*UAS::β_PS_;UAS::α_PS1_*	[32]		**E**
*UAS::Wech-GFP*	[33]		**S**
*UAS::ILK*	[8]		s
*UAS::dPTEN*	[34] Line (2^nd^ chr)		**S**
	[35] Line 31		s
*UAS::dPTEN^C124S^*	[35]		s
*UAS::IRβ_PS_*	*mys^KK100518^*	7D5	**E**
*UAS::IRZasp52*	*Zasp52^GD14816^*	52C4–52C7	**E**
	*Zasp52^KK101276^*		**E**
*UAS::IRparvin*	*Parvin^GD3687^*	18E5–18F1	**S**
	*Parvin^KK102567^*		**S**
*UAS::IRChd64*	*Chd64^GD1212^*	64A6;A7	**E**
*UAS::IREip93F*	*Eip93F^GD4449^*	93F14	**E**/gl
*UAS::IRXrp1*	*Xrp1^KK104477^*	91D3;D5	**S**
*Df(3R)ED2*	BL-6962	91A5;91F1	**S**
*Df(3L)Exel6119*	BL-7598	70B2;70C2	**S**
*Df(3R)e-GC3*	BL-5798	93C6;94A4	**E**

**Table key**. **E**: Enhancement; e: mild enhancement; **S**: Suppression; s: mild suppression. Modifiers phenotype when expressed under longGMR alone; mr: mild rough-eye, gl:glassy.

Cadherin downregulation and initiation of the epithelial-mesenchymal transition (EMT) are typical features of cells acquiring invasive properties [22]. Although the majority of the cells expressing UAS::Parvin-GFP were extruded on the basal side of the posterior wing epithelium, the amount and pattern of cadherin distribution was unaffected in the remaining cells that maintained their plasma membrane in the apical side of the disc (Figure 5F–G). We therefore concluded that Parvin overexpression did not trigger EMT in the wing epithelium.

**Figure 15:**
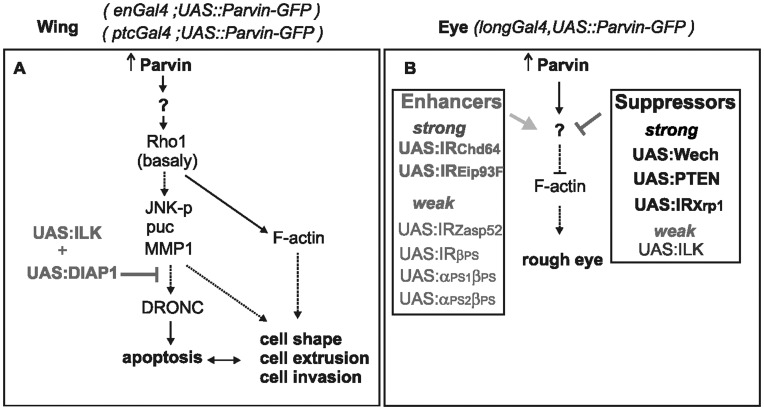
Model of the genetic interactions of Parvin in the wing and eye. (A) The consequences of elevated levels of Parvin in the wing epithelium based on data from this and previous studies [25], [26]. Parvin induces the upregulation of protein levels of Rho1 basally that leads to activation of the JNK pathway resulting in apoptosis. The abnormal levels of Rho1 could also affect the organization of F-actin leading to changes in cell shape, cell exclusion and cell invasion. Collectively these events can also enhance apoptosis and vice versa, as indicated by the double arrow. The coexpression of both ILK and DIAP1 suppresses the activation of DRONC and ameliorates cell exclusion. (B) The consequences of Parvin overexpression in the developing eye that lead to disruption of F-actin basally and the formation of a rough eye. This phenotype is suppressed or enhanced by overexpression or knockdown of specific genes.

### Features of the Wing Epithelial Cells Expressing High Levels of Parvin-GFP

Upon Parvin-GFP overexpression in the posterior wing compartment, we noticed a mosaic expression of the transgene (Figures 2, 3, 5, 6). Certain areas within the *enGal4* domain, notably in the hinge and notum, were not labelled for Parvin-GFP although they properly expressed Engrailed and retained their posterior compartment identity (Figure 6A–C). In these cells *enGal4* was able to direct expression of a UAS::ABDMoesin-RFP transgene (Figure 6D–F), suggesting that the lack of Parvin-GFP labeling was not due to defective *enGal4* activity. In aggrement with this, when we probed wing imaginal discs with an antibody against Parvin, we found that in certain areas of the epithelium, where Parvin-GFP was undetectable, high levels of the protein were present as expected due to overexpression (Fig. 7A–D). In some of these areas, we found apoptotic cells with basaly located pyknotic nuclei (Fig. 7C). We concluded that in these cells, GFP could be destabilized due to undergoing apoptosis. In other areas of the disc undetectable Parvin-GFP was correlated with high density of nuclei (Fig. 7B). That could reflect newly proliferating cells contributing in the regeneration of the damaged epithelium [23]. Therefore in these cells, GFP may have not matured yet to obtain fluorescent properties.

### High Levels of Parvin Induce Cell Delamination, Cell Shape Changes and Disorganization of F-actin on the Basal Side of the Wing Epithelium

To address whether the mosaic expression of Parvin-GFP within the wing epithelium and cell delamination along the apicobasal axis of the blade were also accompanied by changes in cell shape, we examined the F-actin cytoskeleton organization. In the most apical area of the wing blade the tissue was folded and the posterior compartment appeared shrunken, while the amount and distribution of F-actin cortically appeared normal. From the location of the nuclei in optical cross-sections -obtained at the region between the dorsal-ventral boundary in the middle of the wing poutch- it was evident that cells were shorter (Figure 8A, G, I). In the middle area of the wing disc, cells expressing Parvin-GFP occupied a larger region of the wing blade whereas cell shape, as it was highlighted by F-actin, was similar to the flanking cells in the anterior compartment that did not express high levels of full-length Parvin-GFP (Figure 8C). On the basal side, Parvin-GFP expressing cells occupied almost the entire posterior wing blade, but they were missing from the regions flanking the wing margin (Fig. 8E). The organization of F-actin basaly was completely disrupted (Fig. 8E, E′). Actin filaments were accumulated ectopically in some areas of the wing poutch cells and were missing from others. The observed gaps containing pyknotic nuclei, indicating areas of dead delaminated cells (Fig. 8E, E′′), in accordance with previous studies describing the basal extrusion of dead cells in the wing epithelium [23], [24]. As consequence of the damaged epithelium, the basal cell periphery appeared enlarged and irregularly shaped (Figure 8E′). Coexpression of either ILK or DIAP1 with Parvin-GFP noticeably improved the cell delamination at the basal side (Figure 9), whereas simultaneous coexpression of both ILK and DIAP1 further improved cell extrusion, as was evident from the reduced number of pyknotic nuclei accumulated basaly (Figure 8B, D, F). However, in the wing blade F-actin organization was only modestly ameliorated by coexpression of both ILK and DIAP1. Actin filaments instead of decorating the outline of the cell, extended to the periphery and remained tangled resulting in a disordered meshwork pattern (Figure 8B–B′, D–D′, F–F′, H, I). To test whether the disorganised F-actin is correlating with abnormal cell-matrix adhesion mediated by increased levels of Parvin-GFP rather than being a consequence of apoptosis, we examined the distribution of integrins and lamininA in discs coexpressing ILK and DIAP1, where apoptosis was rescued (Fig. 2). No improvement in the abnormal organization of either integrin or lamininA basaly in the wing epithelium was observed (Figure 10A–B). Thus, defects in integrin-mediated adhesion in the basal side of the epithelium upon Parvin-GFP overexpression is not a consequence of Parvin-induced apoptosis, but rather a distinct effect that correlates with abnormalities in the organization of actin cytoskeleton.

### Parvin Overexpression Induces Up-regulation of Rho1 at the Basal Side of the Epithelium

The Parvin-GFP induced alterations in the wing epithelium were highly reminiscent of those observed upon Rho1 overexpression [25], [26]. We found that cells overexpressing Parvin-GFP triggered a substantial increase in Rho1 protein levels, mostly on the basal side (Figure 11A, E), whereas Rho1 accumulation increased only modestly in the middle and in most apical areas of the epithelium (Figure 11B, E). However, the increase in Rho1 levels represented a distinct effect, different from Parvin-induced apoptosis, because elevated Rho1 levels were unaffected, even when both ILK and DIAP1 were coexpressed (Figure 11C–E). Diaphanus (Dia) is one of the main Rho1 downstream effectors. However, as previously found in wing discs [25], Rho1 elevation did not coincide with increased Dia levels upon Parvin-GFP overexpression (Figure 12).

### Parvin Overexpression Disrupts F-actin Stress Fibers in the Pigment Cells of the Pupal Retina

Parvin overexpression by *longGMRGal4* caused a rough eye phenotype (Table 1, Figure 1). This Gal4 driver is expressed in all cell types of the eye (pigment, cone and photoreceptor cells) [27]. The *elavGal4* and *sevGal4* drivers that limit expression of Parvin-GFP to only the photoreceptor [28], or specific photoreceptor and cone cells [29], respectively, did not cause any eye roughening (Table 1). Thus, the rough-eye phenotype is most likely caused by overexpression of Parvin in the pigment cells. Several morphogenetic defects during eye development could result in final eye roughening [30]. We therefore examined the organization of F-actin in both 3^rd^ instar larvae and at 75% of pupal development (p.d). The later developmental stage was selected because in the retinal floor, F-actin displays a highly ordered structure of stress fibers within the pigment cells encircling the cone cells [31]. We did not find any defects in F-actin organization in the eye imaginal discs from 3^rd^ instar larvae (data not shown). In contrast, when we examined retinas from late pupae, we found complete disorganization of actin stress fiber arrays in the retina floor, whereas retinas expressing the truncated UAS::Parvin^ΔCH2^-GFP form appeared normal (Figure 13A, B). Thus, in the pupal retina Parvin-GFP overexpression severely disrupted F-actin stress fiber organization in the basal side of the pigment cells, similar to the wing epithelium phenotype.

### Genetic Interactors of Parvin in the Eye

The homozygous *longGMRGal4* flies had wild type-like eye morphology when kept at 25°C (Figure 14A). In contrast, flies overexpressing Parvin-GFP under *longGMRGal4* displayed distorted ommatidia and mild rough eyes (Figure 14B). This phenotype was sensitive to the copy number of both *longGMRGal4* and UAS::Parvin-GFP transgenes (data not shown), and could therefore be exploited to identify genetic suppressors and enhancers of UAS::Parvin-GFP overexpression-induced rough-eye.

We investigated individual coexpression of a panel of selected available UAS genes in the eye. Loss of function mutations in both α_PS1_ and β_PS_ integrin subunits affect the F-actin pattern at the basal surface of the eye retina in late pupae [31]. We hypothesized that if Parvin-GFP overexpression compromised integrin-containing adhesion sites, as we found in the wing epithelium, then coexpression of an integrin heterodimer (α_PS1_β_PS_ or the α_PS2_β_PS_) would ameliorate the Parvin-induced phenotype [32]. In contrast, we found that elevated levels of either of the two coexpressed integrin heterodimers mildly enhanced the UAS::Parvin-GFP induced rough-eye phenotype (Table 3). However, because the levels of integrin expression are not accurately controlled in this experimental setting it is plausible that high levels of integrin expression could not reverse the Parvin-induced rough eye phenotype. Thus, we concluded that a tight balance of the intracellular amount of integrins appears to be rather crucial for the proper eye development. UAS::ILK weakly suppressed the Parvin induced rough-eye phenotype (Table 3). Surprisingly, coexpression of UAS::Wech-GFP, an ILK binding protein [33], completely suppressed the phenotype (Figure 14C). The morphology and organization of the ommatidia remained intact when UAS::Wech-GFP was overexpressed alone under *longGMRGal4* (Table 3). A strong suppressive effect was also achieved by coexpression of UAS::PTEN [34] (Figure 14E). In contrast, the catalytically inactive PTEN^C124S^ mutant [35] was a poor suppressor, suggesting that enzymatically active PTEN is required to modulate Parvin effects.

Next we conducted a dominant-modifier screen using chromosomal deficiency lines of the third chromosome (Bloomington kit) that covered almost 40% of the fly genome. In the first round we tested 111 deficiencies covering almost the entire 3^rd^ chromosome and found 4 suppressors and 12 enhancers. We further narrowed down three genomic regions that were dominant modifiers (present as just one copy) of Parvin-induced rough-eye. The cytogenetic regions encompassing 70B2;70C2 {*Df(3L)Exel6119*} and 91A5;91F1 {*Df(3R)ED2*} were identified as strong suppressors (Figure 14G, I), whereas the region 93C6;94A4 {*Df(3R) e-GC3*} was an enhancer (Figure 14D). To identify candidate genes, we used individual knock-down of 391 specific genes in the eye utilizing UAS::IR lines [36] for the majority of the genes located in the identified genomic regions. No candidate gene was identified for the dominant suppressive effect of 70B2; 70C2. Knock-down of *Xrp1* (*CG17836*, 91D3-D5) within *Df(3R)ED2* was equally efficient at suppressing the removal of one copy of the genomic region 91A5; 91F1 (Figure 14K). In addition, knock-down of *Eip93F* (*CG18389*, 93F14) located in the genomic region 93C6; 94A4 enhanced the rough-eye, similarly to *Df(3R) e-GC3* (Figure 14F). Lastly, knock-down of genes encoding βPS integrin, Zasp52, and the transgelin homolog Chd64 (*CG14996*) all enhanced the Parvin-induced rough-eye phenotype (Figure 14H, J, L).

## Discussion

Parvin proteins are highly conserved and participate in the assembly and function of the integrin adhesome [3], [6]. Here we employed the *UAS/Gal4* system to investigate additional functions of Parvin upon overexpression in a tissue specific manner and to identify novel genetic interactions in the wing and the eye (Figure 15).

We showed that *Drosophila* Parvin promoted apoptosis when overexpressed *in vivo*, similar to mammalian β-Parvin in HeLa cells [11]. Expression of β-Parvin in breast cancer cells was recently shown to inhibit tumor progression and cell proliferation [7] suggesting that our study of the cellular and molecular changes associated with Parvin overexpression in *Drosophila* may be relevant to cancer pathology. At the cellular level we demonstrated that overexpressed Parvin induced alterations in the organization of the actin cytoskeleton, disruption of cell-matrix adhesion, cell invasion and cell delamination. Mechanistically, we showed that overexpressed Parvin causes JNK activation and enhanced MMP1 levels. We also revealed a functional link between Parvin and subcellular distribution of Rho1. Interestingly, we showed that these Parvin-induced signaling effects are not dependent on its interaction with ILK.

Among the three counterparts of the ILK/PINCH/Parvin-complex, only overexpression of full-length Parvin induced ectopic apoptosis and excessive lethality in the larval and pupae developmental stages [6]. Nevertheless, in the wing imaginal discs overexpression of other components of the integrin adhesome such as tensin and paxillin also result in apoptosis and lethality, including activation of the JNK pathway and modulation of Rho1 activity, respectively [37], [38]. We showed that overexpression of Parvin increases Rho1 protein levels predominantly at the basal side of the wing epithelium, although loss of Parvin did not cause a reciprocal reduction of Rho1 levels [5], [6]. Given the previous reports that mammalian Parvins interact with two regulators of the small GPTases family, the GEF αpix and the CdGAP respectively [39], [40], one hypothesis would be that high levels of Parvin sequester these factors and interfere with their interaction with Rho1. As a consequence, Rho1 is released from the apicolateral side where normally is enriched [41]. The elevated Rho1 levels in the basal compartment of the epithelium could explain the formation of ectopic actin accumulation in accordance with previous studies [42]. As already described Rho1 is able to induce JNK-dependent apoptosis and F-actin organization defects in the wing epithelia cells [25], [26]. Therefore, it is plausible that the elevated JNK activity observed upon Parvin overexpression is caused by aberrant elevation of Rho1 basaly. Taken our findings together, we propose that Parvin-induced cellular defects in the wing epithelia are mediated by increased levels of Rho1, however, we cannot rule out a putative role of additional unidentified factors that are activated downstream of Parvin independently of Rho1.

We recently showed that coexpression of ILK together with Parvin-GFP in the mesoderm is sufficient to completely rescue Parvin-induced lethality and control Parvin subcellular localization [6], suggesting that coupling of Parvin to ILK could have a protective effect in epithelia viability. We performed rescue experiments to investigate whether Parvin function in the wing epithelium is mechanistically linked to its interaction with ILK, by coexpressing Parvin with ILK. Expression of ILK alone did not completely rescue the dominant effects of Parvin overexpression in the developing wing epithelia, had a mild suppressive effect on the rough eye phenotype and did not change the subcellular distribution of Parvin-GFP in the wing epithelial cells. Both the JNK activity and the increase in Rho1 protein levels were also not affected by ILK coexpression. Even when high levels of ILK are present, the putative interaction of Parvin with GTPase regulators is not disturbed and the imbalance of Rho1 subcellular distribution is maintained. That is not unexpected given that both αpix and CdGAP interact with the N-terminus region of Parvin, whereas ILK bind on the C-terminus. These findings demonstrate that the functional interplay between Parvin and ILK depends on the cell context and that Parvin interacts with other proteins and perform additional roles. In addition to functioning as a structural element of the integrin-actin link, it also acts as a dosage dependent modulator of actin cytoskeleton organization and cell homeostasis in the developing epithelia, via modulating the subcellular distribution of Rho1.

Because overexpression of Parvin caused extensive apoptosis in the wing epithelium, to mechanistically uncouple the Parvin-induced cellular defects from Parvin-induced apoptosis, we performed rescue experiments by coexpressing Parvin and DIAP1, which blocks apoptosis by inhibiting both the initiator caspase DRONC and the effector caspases DriCe and Dcp-1 [43]. DIAP1 alone did not efficiently suppress the cellular defects of Parvin in the wing. Both ILK and DIAP1 had to be coexpressed to completely rescue the lethality, presumably by coupling the reduction of excessive cytoplasmic Parvin by ILK and the inhibition of DRONC-mediated apoptosis by DIAP1. Coexpression of ILK and DIAP1 rescue both cell apoptosis and cell extrusion in the wing poutch cells, but not in the hinge and notum. These findings were not entirely unexpected, given previous documentation of regional differences within the wing imaginal disc regarding the differential requirement of actin regulators for epithelial integrity [44]. However, in consistence with our results from ILK rescue experiments, coexpression of DIAP1 or both ILK and DIAP1 did not ameliorate either the irregular organization of F-actin or the disorganized integrin-matrix adhesion sites and did not change the elevated levels of Rho1 in the basal side of the wing epithelium. These results demonstrate that the Parvin-induced cellular defects are not a simple consequence of apoptosis, but rather a distinct feature of Parvin function.

Overexpression of Parvin in the eye generated a rough eye phenotype. At the cellular level the basal actin cytoskeleton in the eye retina was severely disrupted, suggesting that a cause of the abnormal eye development could be initiated by abnormalities in the cell shape of pigment cells, as in the case of the wing epithelium. Because the Parvin-induced eye phenotype was sensitive to the copy number of Parvin transgenes and to temperature, we performed a modifier screen to uncover novel genetic interactors. We found that elevated levels of Wech and PTEN antagonized the Parvin-induced dominant effects in the developing eye and completely suppressed the rough eye phenotype, whereas high levels of ILK had only minimal suppression activity.

Wech is an ILK binding protein and it is not clear why it could suppress Parvin-induced dominant defects at elevated levels [33] rather than ILK itself, which directly binds to Parvin and rescues lethality completely in the mesoderm [6] and significantly in *enGal4* expressing cells. The lack of data regarding Wech function in the eye, preclude further analysis at this point. The second surprising result of our study was the ability of high levels of PTEN to suppress the rough eye phenotype induced by Parvin overexpression. UAS::PTEN overexpression under *GMRGal4* has been reported to induce a rough-eye phenotype by inhibiting cell-cycle progression in proliferating cells and inducing apoptosis in a cell-context dependent manner [35], [45]. In our experiments expression of the same UAS::PTEN lines obtained from two different donors [35], [45] did not result in eye roughening. One possible explanation could be the use of *longGMRGal4* (Bloomington #8506) in our experiments, because previous studies drove expression of UAS::PTEN with *GMRGal4* [46]. In addition, previous reports suggested that expression by *longGMRGal4* driver in the developing eye follows a more strict pattern in the photoreceptor cells [47]. Taken together our data and previous reports, we speculate that Parvin and PTEN have antagonistic functions within the eye epithelium and coexpression of both proteins counterbalance their induced dominant effects upon overexpression. Currently we do not have sufficient data to point a specific pathway that could be modified by Parvin and PTEN and leads to rough eye phenotype. However, the recent report that Parvin is associated with PKB [48] together with previous data suggesting that Parvin may facilitates the recruitment of PKB at plasma membrane [10], suggests that Parvin could antagonized the negative effects of PTEN on PKB activation by reducing PIP3 levels [49].

The third suppressor gene we found was *Xrp1*. *Xrp1* contains an AT-hook motif that is found in nuclear proteins with DNA binding activity. Currently, we lack sufficient knowledge to speculate on putative functional interaction between Parvin-induced signaling and nuclear activity. However, previous studies on *Xrp1* point on its role as a p53-dependent negative regulator of cell proliferation following genotoxic stress [50]. Among the genes that enhanced the Parvin-induced rough eye were all of the integrin subunits known to be expressed in the eye, including α_PS1_, α_PS2_ and β_PS_ [31], the cytoskeletal regulators ZASP52 [51] and the transgelin homolog encoded by CG14996 of unknown function.

In conclusion, our findings revealed novel cell context-dependent roles for Parvin in the whole organism. Besides its known function as a structural component of the IPP-complex that mediate the integrin-actin link, we demonstrated that Parvin can also affect cell-matrix adhesion, organization of actin cytoskeleton and cell homeostasis, by regulating Rho1 and JNK levels in an ILK-independent manner. These findings are relevant to situations where cell homeostasis is altered ranging from the physiological renewal of tissues to cancer pathology. In addition, our modifier genetic screen revealed novel interactors that affect Parvin function in a living organism. Our *in vivo* data provide the first insight into genetic circuits influenced by Parvin and offer a framework for additional detailed studies to elucidate how these genetic networks interact.

## References

[1] Bokel C, Brown N (2002) Integrins in development: moving on, responding to, and sticking to the extracellular matrix. Dev Cell 3: 311–321. 12361595 10.1016/s1534-5807(02)00265-4

[2] Geiger B, Yamada KM (2011) Molecular Architecture and Function of Matrix Adhesions Cold Spring Harb Perspect Biol. 3: a005033. 10.1101/cshperspect.a005033 PMC3101841 21441590

[3] Sepulveda JL, Wu C (2006) The parvins. Cell Mol Life Sci 63: 25–35. 16314921 10.1007/s00018-005-5355-1 PMC2792345

[4] Wickstrom SA, Lange A, Montanez E, Fassler R (2010) The ILK/PINCH/parvin complex: the kinase is dead, long lived the pseudokinase! EMBO J. 29: 281–291. 10.1038/emboj.2009.376 PMC2824469 20033063

[5] Montanez E, Wickström SA, Altstätter J, Chu H, Fässler R (2009) Alpha-parvin controls vascular mural cell recruitment to vessel wall by regulating RhoA/ROCK signalling. EMBO J 28: 3132–3144. 19798050 10.1038/emboj.2009.295 PMC2771098

[6] Vakaloglou KM, Chountala M, Zervas CG (2012) Functional analysis of parvin and different modes of IPP-complex assembly at integrin sites during Drosophila development. J Cell Sci 125: 3221–3232. 22454516 10.1242/jcs.102384

[7] Johnstone CN, Mongroo PS, Rich AS, Schupp M, Bowser MJ, et al (2008) Parvin-beta inhibits breast cancer tumorigenicity and promotes CDK9-mediated peroxisome proliferator-activated receptor gamma 1 phosphorylation. Mol Cell Biol 28: 687–704. 17998334 10.1128/MCB.01617-06 PMC2223422

[8] Zervas CG, Gregory SL, Brown NH (2001) Drosophila integrin-linked kinase is required at sites of integrin adhesion to link the cytoskeleton to the plasma membrane. J Cell Biol 152: 1007–1018. 11238456 10.1083/jcb.152.5.1007 PMC2198807

[9] Gutzeit HO, Eberhardt W, Gratwohl E (1991) Laminin and basement membrane-associated microfilaments in wild-type and mutant Drosophila ovarian follicles. J Cell Sci 100: 781–788. 1814932 10.1242/jcs.100.4.781

[10] Fukuda T, Guo L, Shi X, Wu C (2003) CH-ILKBP regulates cell survival by facilitating the membrane translocation of protein kinase B/Akt. J Cell Biol 160: 1001–1008. 12654898 10.1083/jcb.200212113 PMC2172761

[11] Zhang Y, Chen X, Tu Y, Wu C (2004) Distinct roles of two structurally closely related focal adhesion proteins, a-parvins and b-parvins, in regulation of cell morphology and survival. J Biol Chem 279: 41695–41705. 15284246 10.1074/jbc.M401563200

[12] Brand AH, Perrimon N (1993) Targeted gene expression as a means of altering cell fates and generating dominant phenotypes. Development 118: 401–415. 8223268 10.1242/dev.118.2.401

[13] Fan YaB, A. (2010 ) The cleaved-Caspase-3 antibody is a marker of Caspase-9-like DRONC activity in Drosophila. Cell Death Differ 17: 534–539. 19960024 10.1038/cdd.2009.185 PMC2822068

[14] Adachi-Yamada T, Fujimura-Kamada K, Nishida Y, Matsumoto K (1999) Distortion of proximodistal information causes JNK-dependent apoptosis in Drosophila wing. Nature 400: 166–169. 10408443 10.1038/22112

[15] Agnès F, Suzanne M, Noselli S (1999) The Drosophila JNK pathway controls the morphogenesis of imaginal discs during metamorphosis. Development 126: 5453–5462. 10556069 10.1242/dev.126.23.5453

[16] Wang SL, Hawkins CJ, Yoo SJ, Müller HA, Hay BA (1999) The Drosophila caspase inhibitor DIAP1 is essential for cell survival and is negatively regulated by HID. Cell 98: 453–463. 10481910 10.1016/s0092-8674(00)81974-1

[17] Page-McCaw A, Serano J, Sante JM, Rubin GM (2003) Drosophila matrix metalloproteinases are required for tissue remodeling, but not embryonic development. Dev Cell 4: 95–106. 12530966 10.1016/s1534-5807(02)00400-8

[18] Brown NH, Gregory SL, Rickoll WL, Fessler LI, Prout M, et al (2002) Talin is essential for integrin function in Drosophila. Dev Cell 3: 569–579. 12408808 10.1016/s1534-5807(02)00290-3

[19] Domínguez-Giménez P, Brown N, Martín-Bermudo M (2007) Integrin-ECM interactions regulate the changes in cell shape driving the morphogenesis of the Drosophila wing epithelium. J Cell Sci 120: 1061–1071. 17327274 10.1242/jcs.03404

[20] Breitsprecher D, Kiesewetter AK, Linkner J, Vinzenz M, Stradal TE, et al (2011) Molecular mechanism of Ena/VASP-mediated actin-filament elongation. EMBO J 30: 456–467. 21217643 10.1038/emboj.2010.348 PMC3034019

[21] Becam I, Rafel N, Hong X, Cohen SM, Milán M (2011) Notch-mediated repression of bantam miRNA contributes to boundary formation in the Drosophila wing. Development 138: 3781–3789. 21795284 10.1242/dev.064774

[22] Thiery JP, Acloque H, Huang RY, Nieto MA (2009) Epithelial-mesenchymal transitions in development and disease. Cell 139: 871–890. 19945376 10.1016/j.cell.2009.11.007

[23] Bergantiños C, Corominas M, Serras F (2010) Cell death-induced regeneration in wing imaginal discs requires JNK signalling. Development 137: 1169–1179. 20215351 10.1242/dev.045559

[24] Li W, Baker N (2007) Engulfment is required for cell competition. Cell 129: 1215–1225. 17574031 10.1016/j.cell.2007.03.054

[25] Neisch AL, Speck O, Stronach B, Fehon RG (2010) Rho1 regulates apoptosis via activation of the JNK signaling pathway at the plasma membrane. J Cell Biol 189: 311–323. 20404112 10.1083/jcb.200912010 PMC2856900

[26] Vidal M, Larson DE, Cagan RL (2006) Csk-deficient boundary cells are eliminated from normal Drosophila epithelia by exclusion, migration, and apoptosis. Dev Cell 10: 33–44. 16399076 10.1016/j.devcel.2005.11.007

[27] Grzeschik NA, Knust E (2005) IrreC/rst-mediated cell sorting during Drosophila pupal eye development depends on proper localisation of DE-cadherin Development. 132: 2035–2045. 10.1242/dev.01800 15788453

[28] Lin DMaG, C S (1994) Ectopic and increased expression of Fasciclin II alters motoneuron growth cone guidance. Neuron 13: 507–523. 7917288 10.1016/0896-6273(94)90022-1

[29] Tomlinson A, Bowtell DD, Hafen E, Rubin GM (1987) Localization of the sevenless protein, a putative receptor for positional information, in the eye imaginal disc of Drosophila. Cell 51: 143–150. 3115593 10.1016/0092-8674(87)90019-5

[30] Cagan R (2009) Principles of Drosophila eye differentiation. Curr Top Dev Biol 89: 115–135. 19737644 10.1016/S0070-2153(09)89005-4 PMC2890271

[31] Longley RL, Ready DF (1995) Integrins and the development of three-dimensional structure in the *Drosophila* compound eye. Dev Biol 171: 415–433. 7556924 10.1006/dbio.1995.1292

[32] Martin-Bermudo MD, Dunin-Borkowski OM, Brown NH (1997) Specificity of PS integrin function during embryogenesis resides in the a subunit extracellular domain. EMBO 16: 4184–4193. 10.1093/emboj/16.14.4184 PMC1170044 9250662

[33] Löer B, Bauer R, Bornheim R, Grell J, Kremmer E, et al (2008) The NHL-domain protein Wech is crucial for the integrin-cytoskeleton link. Nat Cell Biol 10: 422–428. 18327251 10.1038/ncb1704

[34] Staveley BE, Ruel L, Jin J, Stambolic V, Mastronardi FG, et al (1998) Genetic analysis of protein kinase B (AKT) in *Drosophila* . Current Biology 8: 599–602. 9601646 10.1016/s0960-9822(98)70231-3

[35] Huang H, Potter CJ, Tao W, Li DM, Brogiolo W, et al (1999) PTEN affects cell size, cell proliferation and apoptosis during Drosophila eye development. Development 126: 5365–5372. 10556061 10.1242/dev.126.23.5365

[36] Dietzl G, Chen D, Schnorrer F, Su KC, Barinova Y, et al (2007) A genome-wide transgenic RNAi library for conditional gene inactivation in Drosophila. Nature 448: 151–156. 17625558 10.1038/nature05954

[37] Lee SB, Cho KS, Kim E, Chung J (2003) blistery encodes Drosophila tensin protein and interacts with integrin and the JNK signaling pathway during wing development. Development 130: 4001–4010. 12874122 10.1242/dev.00595

[38] Chen GC, Turano B, Ruest PJ, Hagel M, Settleman J, et al (2005) Regulation of Rho and Rac signaling to the actin cytoskeleton by paxillin during Drosophila development. Mol Cell Biol 25: 979–987. 15657426 10.1128/MCB.25.3.979-987.2005 PMC544021

[39] Mishima W, Suzuki A, Yamaji S, Yoshimi R, Ueda A, et al (2004) The first CH domain of affixin activates Cdc42 and Rac1 through alphaPIX, a Cdc42/Rac1-specific guanine nucleotide exchanging factor. Genes Cells 9: 193–204. 15005707 10.1111/j.1356-9597.2004.00717.x

[40] LaLonde DP, Grubinger M, Lamarche-Vane N, Turner CE (2006) CdGAP Associates with Actopaxin to Regulate Integrin-Dependent Changes in Cell Morphology and Motility. Curr Biol 16: 1375–1385. 16860736 10.1016/j.cub.2006.05.057

[41] Widmann TJ, Dahmann C (2009) Dpp signaling promotes the cuboidal-to-columnar shape transition of Drosophila wing disc epithelia by regulating Rho1. J Cell Sci 122: 1362–1373. 19366729 10.1242/jcs.044271

[42] Speck O, Hughes SC, Noren NK, Kulikauskas RM, Fehon RG (2003) Moesin functions antagonistically to the Rho pathway to maintain epithelial integrity. Nature 421: 83–87. 12511959 10.1038/nature01295

[43] Fan Y, Bergmann A (2008) Apoptosis-induced compensatory proliferation. The Cell is dead. Long live the Cell! Trends in Cell Biol 18: 467–473. 18774295 10.1016/j.tcb.2008.08.001 PMC2705980

[44] Janody F, Treisman JE (2006) Actin capping protein alpha maintains vestigial-expressing cells within the Drosophila wing disc epithelium. Development 133: 3349–3357. 16887822 10.1242/dev.02511 PMC1544359

[45] Scanga SE, Ruel L, Binari RC, Snow B, Stambolic V, et al (2000) The conserved PI3′K/PTEN/Akt signaling pathway regulates both cell size and survival in Drosophila. Oncogene 19: 3971–3977. 10962553 10.1038/sj.onc.1203739

[46] Kramer JM, Staveley BE (2003) GAL4 causes developmental defects and apoptosis when expressed in the developing eye of Drosophila melanogaster. Genet Mol Res 2: 43–47. 12917801

[47] Ellis MC, O’Neill EM, Rubin GM (1993) Expression of Drosophila glass protein and evidence for negative regulation of its activity in non-neuronal cells by another DNA-binding protein. Development 119: 855–865. 8187644 10.1242/dev.119.3.855

[48] Kimura M, Murakami T, Kizaka-Kondoh S, Itoh M, Yamamoto K, et al (2010) Functional molecular imaging of ILK-mediated Akt/PKB signaling cascades and the associated role of beta-parvin. J Cell Sci 123: 747–755. 20164304 10.1242/jcs.052498

[49] Radimerski T, Montagne J, Hemmings-Mieszczak M, Thomas G (2002) Lethality of Drosophila lacking TSC tumor suppressor function rescued by reducing dS6K signaling. Genes Dev 16: 2627–2632. 12381661 10.1101/gad.239102 PMC187466

[50] Akdemir F, Christich A, Sogame N, Chapo J, Abrams JM (2007) p53 directs focused genomic responses in Drosophila. Oncogene 26: 5184–5193. 17310982 10.1038/sj.onc.1210328

[51] Jani K, Schöck F (2007) Zasp is required for the assembly of functional integrin adhesion sites. J Cell Biol 179: 1583–1597. 18166658 10.1083/jcb.200707045 PMC2373490

